# The Prevalence of Asymptomatic Infections with Tick-Borne Encephalitis Virus and Attitude towards Tick-Borne Encephalitis Vaccine in the Endemic Area of Northeastern Poland

**DOI:** 10.3390/vaccines10081294

**Published:** 2022-08-10

**Authors:** Ewa Bojkiewicz, Kacper Toczylowski, Sambor Grygorczuk, Beata Zelazowska-Rutkowska, Justyna Dunaj, Agnieszka Zebrowska, Piotr Czupryna, Anna Moniuszko-Malinowska, Artur Sulik

**Affiliations:** 1 Department of Pediatric Infectious Diseases, Medical University of Bialystok, Waszyngtona 17, 15-274 Bialystok, Poland; 2 Department of the Infectious Diseases and Neuroinfections, Medical University of Bialystok, Żurawia 14, 15-540 Bialystok, Poland; 3 Department of Pediatric Laboratory Diagnostics, Medical University of Bialystok, Waszyngtona 17, 15-274 Bialystok, Poland; 4 Regional Centre of Transfusion Medicine in Bialystok, Marii Skłodowskiej-Curie 23, 15-950 Bialystok, Poland

**Keywords:** tick-borne encephalitis virus, tick-borne encephalitis, seroprevalence, blood donors, TBEV vaccination, Poland

## Abstract

In Poland, tick-borne encephalitis (TBE) vaccination rate is low despite high incidence of severe infections with TBE virus (TBEV). However, infection with TBEV can be asymptomatic or mild, which makes the total number of cases difficult to assess. We aimed at assessing asymptomatic TBEV infections and describing attitudes towards the TBE vaccine. We studied 298 healthy adult blood donors and 180 children from the TBE endemic area of northeastern Poland for the presence of anti-TBEV IgG antibodies. We also surveyed a separate cohort of 444 adults. Thirty-eight blood donors (13%) and 38 survey respondents (9%) reported a history of a prior anti-TBEV vaccination. Forty respondents (9%) reported vaccinating their child in the past. Fourteen unvaccinated blood donors (5%) and four children (2%) were seropositive for specific anti-TBEV antibodies, suggesting a history of an undiagnosed TBEV infection. In the surveyed cohort, 130 (32%) expressed their intention to be vaccinated and 144 (36%) expressed their intention to vaccinate their child. This intention was significantly higher in respondents with a recent tick-bite, a diagnosis of tick-borne disease in a close relative, and in males. Our study shows that asymptomatic TBEV infections are common. The acceptance of TBE vaccine is low, but might be increased by communicating risks associated with tick bites.

## 1. Introduction

Tick-borne encephalitis (TBE) is an acute central nervous system (CNS) infection caused by tick-borne encephalitis virus (TBEV), transmitted to humans mainly by *Ixodes* spp. ticks. The disease is endemic in large areas of the moderate climate zone of Europe and Asia, with over 10,000 cases of the disease reported annually [1,2]. In Poland, the rate of diagnosed cases is 200–300 per year, about half of the cases from Podlaskie voivodeship in northeastern parts of the country, with local annual morbidity ranging from 4.45 to 9.16 per 100,000 inhabitants in 2019–2021 [3]. The estimated ECDC incidence rates for 2012–2016 in Lithuania, Latvia and Estonia were 15.6, 9.5 and 8.7 cases per 100,000 inhabitants, respectively [4]. The reported morality rate caused by the European type of TBE is around 1% and it is comparable in all European countries [5]. The fraction of TBEV-infected *Ixodes ricinus* ticks in this region has been found to be 0.21% and up to 0.42% locally, similar to the rates found in other recognized TBE endemic areas across Europe [6]. According to serological and molecular studies, currently TBEV is the only *Flavivirus* causing CNS infections in Poland [7,8,9,10]. The clinical severity of TBE varies, ranging from asymptomatic infections to severe encephalitis or myelitis, depending on the not fully understood combination of host and pathogen factors [2,11,12]. The CNS involvement is typically preceded by a distinct peripheral phase dominated by unspecific febrile illness. The second, neurologic phase may take the form of meningitis (with fever, meningeal signs and cerebrospinal fluid—CSF—pleocytosis), meningoencephalitis of variable severity or meningoencephalomyelitis. Usually, patients are hospitalized and the diagnosis is established during the second phase [11,13,14]. However, the background IgG-seropositivity found in healthy non-vaccinated persons from the endemic areas is common and has been attributed to a high number of undiagnosed mild or asymptomatic infections evading hospitalization and correct diagnosis [15,16]. It is difficult to determine the most typical presentation of the non-diagnosed cases. It is unclear whether they are truly asymptomatic or simply too mild and too unspecific to prompt diagnostics toward TBE. However, a study by Lotrič-Furlan suggests that unspecific flu-like febrile disease caused by TBEV is rare and most of the unrecognized cases are asymptomatic [17]. The exact rate and presentation of such cases is difficult to assess, but may be important for the proper understanding of both epidemiology and pathogenesis of the disease. Identification of persons with mild TBEV infections would be informative for the study of the disease pathogenesis and would increase knowledge of the predisposing and protective factors, both genetic and environmental.

Nevertheless, the burden of severe TBE cases in endemic areas is considerable [5]. Follow-up studies after TBE infection showed that up to 46% of patients experience incomplete recovery and report neurological sequalae such as cranial nerve paresis, hemiparesis, paraparesis, impaired balance, coordination, memory and concentration, and reduced stress tolerance [5,18]. In Italy it was confirmed that 1.6% of patients were admitted to intensive care unit due to TBE sequalae [19]. Additionally, due to the absence of any etiological treatment and increasing TBE burden estimated with DALYs, which reflect lost years of healthy life as well as mortality directly, this disease is an increasingly worrying public health problem across the world [20]. The WHO recommends immunizing with the TBE vaccine all individuals living in the area where the disease is common. There are four types of TBE vaccines around the world. Two of them are produced in Russia and are based on Far Eastern TBEV strains. Two other vaccines are based on European subtype TBEV strains [5]. Despite the high prevalence of TBEV infections in Poland, the vaccine uptake remains low. According to the Polish National Institute of Public Health, over the period of 10 years, from 2011 to 2020, only 419,408 individuals in Poland have been vaccinated with TBE vaccines, which constitutes around 1.1% of the total population [3]. The recognition of attitudes towards the TBE vaccine would help overcome vaccine hesitancy in the population in this endemic area. Therefore, the aims of this study were to assess the rate of asymptomatic infections by measuring the seroprevalence of anti-TBE antibodies and to evaluate the attitude towards the TBE vaccine in the population of a highly endemic TBE area in northeastern Poland.

## 2. Materials and Methods

### 2.1. The Prevalence of Anti-TBEV IgG

We examined the presence of anti-TBEV IgG antibodies in a serum sample from the venous blood of adults and children with no active TBEV infection. The adult study group consisted of 300 subjects. Adult subjects were heathy blood donors seen at the Regional Centre of Transfusion Medicine in Bialystok, Poland. Active infections, including type C hepatitis, were excluded before a donation, as a part of a standard qualification procedure. Each participant completed a questionnaire, which included questions about a history of tick-borne encephalitis, and a history of a vaccination against TBE. Two subjects did not return the questionnaires and were excluded from the analysis; they both tested negative for anti-TBEV IgG. The final group consisted of 298 patients: 71% men and 29% women, aged from 18 to 63 years (mean 34.1 years). The pediatric group consisted of 180 hospitalized children aged 2–17 years with signs of viral gastroenteritis, whose serum samples were used in research on the epidemiology and pathogenesis of infectious diseases in children in previous studies [21]. All participants included in the study group gave written informed consent to participate. A 1 mL sample of venous blood was drawn on clot, centrifuged and serum frozen to −80 °C and preserved until all the samples were thawed and studied simultaneously. The serologic examination was performed with FSME/TBE ELISA IgG/IgM from Virotech Diagnostics GmbH (Rüsselsheim, Germany), strictly following the manufacturer’s instructions.

### 2.2. Questionnaire

We also performed an anonymous survey in a cohort of 444 adults recruited among parents whose children were hospitalized in the Department of Pediatric Infectious Diseases in Bialystok, Poland. All respondents lived in northeastern Poland, a high-risk area for tick-borne diseases (TBDs). The survey was carried out between December 2019 and September 2021. The survey consisted of four questions describing practices regarding ticks and tick bites, and seven evaluating demographics and exposure to tick bites.

### 2.3. Ethical Considerations

The study was approved by the Ethics Committee of the Medical University in Białystok (approval numbers R-I-002/241/2013, R-I-002/308/2019, R-I-002/489/2019, and APK.002.71.2020).

### 2.4. Statistical Analysis

Descriptive data are presented as frequencies and percentages. The attitude towards the TBE vaccine was evaluated using the binary logistic regression model. In univariate analysis we identified variables associated with a positive attitude towards the TBE vaccine (either a past vaccination or a declaration to receive the vaccine). In the multivariate analysis we included variables identified in the univariate analysis with the *p*-value equal to or below 0.2. A backward stepwise method was used and variables with a significant association were presented in the graphs only. The results of this analysis are shown as odds ratios (OR) and their respective 95% confidence intervals (95% CI). The statistical analysis was performed using TIBCO Software Inc. (2017) Statistica, version 13 (Palo Alto, CA, USA).

## 3. Results

### 3.1. Detection of Anti-TBEV IgG Antibodies

Four subjects, a woman, aged 25, and men, aged 33, 38 and 58, claimed that they had been diagnosed with TBE in the past. None of them tested positive for anti-TBEV IgG.

Thirty eight adult subjects (13%), including 31 men and seven women, aged 20 to 56, reported having received a vaccine against TBEV, including two who reported having received an incomplete scheme (one or two doses only) and one who reported not having received a booster dose for the last 15 years. The fraction of persons reporting vaccination was higher among men (15%) then women (7%). Twenty-six subjects with a history of TBE vaccination (68%) tested positive for specific anti-TBEV IgG. Two patients with incomplete vaccination were seronegative. Interestingly, the patient last vaccinated 15 years before the examination had a strong positive response.

Of 256 subjects reporting neither vaccination against nor being diagnosed with TBE, 14 (5%, seven men and seven women) tested positive. The remaining 242 adult subjects were seronegative. Groups of seropositive and seronegative vaccinated and non-vaccinated subjects did not differ significantly with age.

The pediatric study group consisted of 180 children aged 2–17 (median age 6 years). None of the children were vaccinated with the TBE vaccine, none were presenting symptoms corresponding with TBE, nor were diagnosed with TBE in the past. A total of four children (2%) tested positive.

### 3.2. Survey Evaluating Willingness to Vaccinate against TBE

We surveyed 444 adults (Table 1). The majority of respondents were women (82% 345/419) aged 30–39 (59%, 252/426), with higher education (66%, 281/426), living in urban areas (59%, 251/426). The surveyed cohort is a close proxy of the population in productive age from northeastern Poland. However, due to the sampling approach women under 50 years of age are overrepresented. Sixty-two percent (270/438) of the respondents have been bitten by a tick at least once in their life, while 17% (75/444) of respondents were bitten in the last year. Approximately 32% (139/433) reported that their child has also been bitten by a tick at least once in their life (Table 1). Only 9% (38/424) of the respondents reported a previous vaccination with the TBE vaccine, while 10% (40/413) have vaccinated their children. Among people who were not vaccinated, 31% (121/386) were willing to receive a TBE vaccine, while the majority (43%, 167/386) were unsure whether to vaccinate against TBE and 25% (98/386) presented an anti-vaccine approach. Amidst the parents who have not vaccinated their children, 37% (138/373) were planning to vaccinate their child, 20% (74/373) did not accept the vaccination, while 43% (161/373) were unsure.

**Figure 1:**
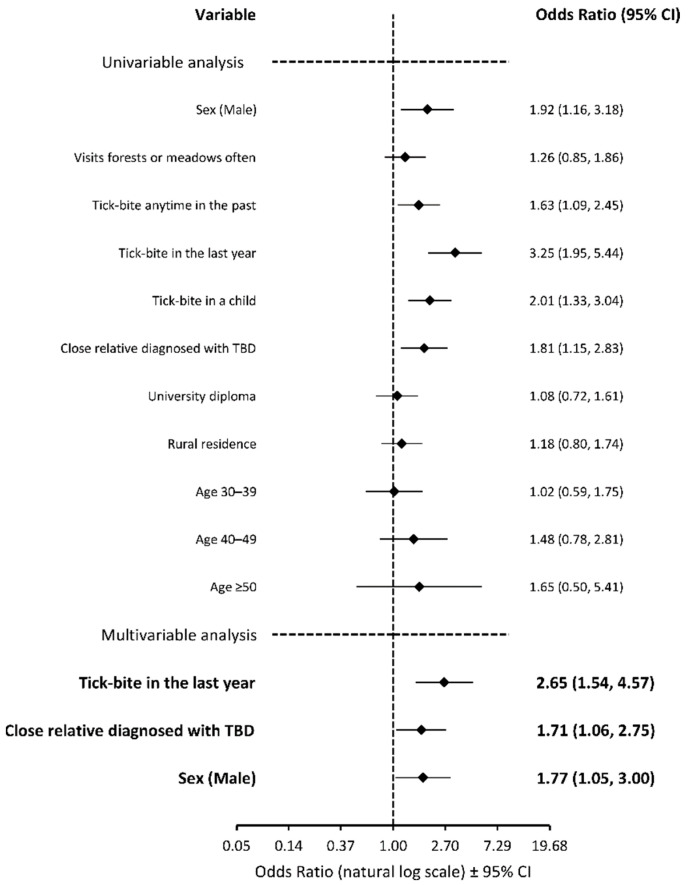
Binominal logistic regression analysis of variables associated with the odds of receiving or declaration to receive the TBE vaccine. The age-related odds were calculated with reference to age under 30 years. Abbreviations: TBD, tick-borne disease.

**Figure 2:**
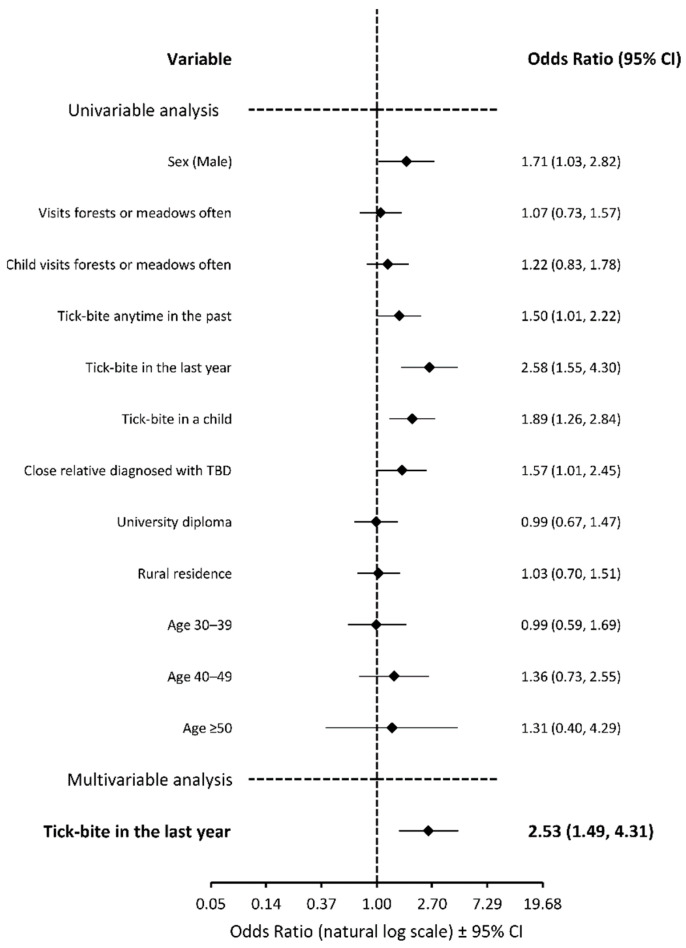
Binominal logistic regression analysis of variables associated with the odds of vaccinating or declaration to vaccinate the respondent’s child with the TBE vaccine. The age-related odds were calculated with reference to age under 30 years. Abbreviations: TBD, tick-borne disease.

In univariate analysis, factors such as a history of a tick bite in the past, a history of a tick bite in a child of a person completing the questionnaire, an occurrence of a tick-borne disease in a close relative, and male gender were associated with a significantly higher likelihood of the respondent (Figure 1) or the respondent’s child (Figure 2) receiving the TBE vaccine. According to the multivariate analysis, the odds of respondents receiving the vaccine were higher in those with a recent tick bite (OR, 2.65; 95% CI, 1.54–4.57; *p* < 0.001), a diagnosis of TBD in a close relative (OR, 1.71; 95% CI, 1.06–2.75; *p* = 0.027), and in males (OR, 1.77; 95% CI, 1.05–3.00; *p* = 0.032). The multivariate analysis also showed that a recent tick bite in a respondent was the only predictive variable for the vaccination of a respondent’s child (OR, 2.53; 95% CI, 1.49–4.31; *p* < 0.001). We have also adopted a similar approach to investigate negative attitudes towards the TBE vaccine (respondents who were not vaccinated in the past and declare not to vaccinate in the future). We found that the younger subjects presented a more negative attitude. Respondents under the age of 40 were less likely to receive the TBE vaccine (OR, 0.40; 95% CI, 0.21–0.76; *p* = 0.006) and to vaccinate their child (OR, 0.30; 95% CI, 0.13–0.67; *p* = 0.004), compared to their older counterparts. Sex, education level, place of residence, and lack of exposure to ticks were not associated with the negative attitude.

## 4. Discussion

In Western and Central Europe, tick-borne encephalitis is endemic and is one of the most important causes of human CNS infections. Currently there is no specific antiviral treatment available for TBE, therefore preventive measures are of prime importance. Vaccines are proven to be highly effective in preventing TBE and the vaccinations have been conducted for more than 30 years [22,23]. Based on the reports from the Polish National Institute of Public Health, the TBE vaccine was administered to around 1.1% of the total Polish population in years 2011–2020 [3]. In our study 13% of blood donors, 9% of the surveyed adults, and 10% of their children were vaccinated with the TBE vaccine. This might reflect an increased interest in the vaccine in the endemic area. According to different researchers, in other endemic European countries, vaccination rates vary between 0 and 40% depending on a region [24,25,26,27]. However, in Latvia, where TBE vaccination has been a part of a national vaccination program since 2007, the vaccination rate exceeds 50% [28,29]. On the other hand, the official vaccine reports might be underestimated because the TBE vaccine is not mandatory in Poland and therefore physicians are not required to report this immunization. Nevertheless, the uptake of the TBE vaccine in Poland is suboptimal. Given the potentially severe clinical course of the disease, the absence of antiviral treatment and the increasing population, ticks vaccination is the mainstay of prevention and control of TBE [18]. Therefore, it is imperative to increase the vaccination rate in populations living in endemic areas. We found that a recent tick bite and a diagnosis of TBD in a close relative were the two main drivers of the decision or intention to receive the TBE vaccine. This probably reflects the higher level of perceived susceptibility to TBDs, which is in line with previous studies, which showed that the more threatened people felt, the more they intended to get vaccinated [30,31,32]. In our research we discovered that the level of education and place of residence did not affect the attitude towards the TBE vaccine. Younger age was noted as a barrier, whereas being male was a promoter of vaccination (Figure 1 and Figure 2). Sociodemographic factors presented a mixed picture of results in previous studies. According to research conducted in Switzerland and Austria, vaccination rates with the TBE vaccine tended to be higher in people under 60 years of age [33]. On the contrary, in Sweden the uptake of the TBE vaccine was higher in those aged over 60 years, compared to younger adults and children [34]. These conflicting research findings suggest that age is not necessarily a consistent motivation of TBE vaccination. The differences are most likely associated with occupation or preferred model of spending free time which varies depending on a country.

In our study, 12 among the 38 subjects reporting the history of vaccination tested negative for specific anti-TBEV IgG, which included two who had not completed the primary vaccination scheme. Interestingly, the patient who received a basic vaccination scheme but did not receive booster doses in the last 15 years obtained a strong humoral response in our study. In general, the seropositivity rate in vaccinated blood donors was rather low compared with literature data. Of note, although the subjects provided additional information regarding the vaccination date and completeness, we were not able to verify the full vaccination history. It was not our aim to verify the vaccination efficacy in a systematic manner, but, taking the above into consideration, our overall results seem consistent with the literature data. Due to the fact that vaccination against TBE has been proven to be highly effective, the rate of seropositivity according to various studies reaches 92–99% and it depends on time since vaccination and the immune status of patients [35]. Although vaccines produced in Europe have high immunogenicity, the persistence of their seroprotection remains in dispute. Studies have shown a gradual decrease in immunocompetence 10 years after the vaccination [36]. This decline is more clearly expressed in older individuals aged over 50 compared to younger people [37].

We should keep in mind that even though the TBE vaccination breakthrough cases are uncommon, they do occur. Several studies performed in Austria, Sweden and Slovenia have shown that between 0.7% to 3.1% of patients get this disease despite complete vaccination. However the majority of cases were observed among patients over 50 years old [38,39]. The severity of observed cases ranges from mild to severe with sometimes long-term neurological sequelae or even a fatal outcome [38,40]. It is also worth noticing that patients with autoimmune diseases, those who are HIV positive and children after thymectomy also present a lowered serological response to the TBE vaccine [41,42].

When it comes to the lack of a specific immune response in all four patients who had TBE diagnosed in the past, it is problematic and difficult to explain only with progressive weakening of the immune response. If these seronegative patients truly had symptomatic TBE in the past, it would mean that the rate of the past asymptomatic infections assessed by IgG serology could have been seriously underestimated. However, as our results are based on a questionnaire filled during the donation procedure, we were unable to verify the diagnosis of TBE in these subjects. The possible confounding diagnoses include viral meningitis of non-TBE etiology, which could be attributed to TBEV in a highly endemic area, and early neuroborreliosis, which is common in the area and could not be differentiated from TBE by the reporting patients.

Five percent of adults and 2% of children who had never been diagnosed with TBE or vaccinated against it tested positive for the specific anti-TBEV IgG. There were no previous TBEV seroprevalence studies conducted among unvaccinated individuals living in a TBE endemic area in Poland. According to the latest data from Norway, the phenomenon of TBEV IgG seroprevalence in the general population ranges from 0.4% up to 1.4% [43,44]. However, among high-risk populations living in endemic areas the seroprevalence rates are substantially higher, ranging from 4% up to 22% depending on the investigated area, which is consistent with our results [45]. No data are available on the seroprevalence of TBEV IgG antibodies in children. Our results show that seroprevalence in children was lower than in adults. This is consistent with lower rates of symptomatic disease in children across Europe [46]. In our previous study conducted on the same area, the annual incidence of neuroinfectious caused by TBEV in children was estimated to be lower compared to the general population [47]. Surprisingly, in Serbia the seroprevalence of TBEV IgG was higher in children than in adults and reached 18%, probably reflecting differences in exposures to tick-bites between populations [48]. TBEV seems to be the only circulating Flavivirus in Poland as our region is not endemic for dengue, yellow fever or Zika. The study performed by Piotr Czupryna et al. in 2014 found no evidence of West Nile RNA in CSF collected from patients with lymphocytic meningitis in Podlaskie voivodship [8]. What is more, the study conducted by the Department of Poultry Diseases, National Veterinary Research Institute on 1912 Polish birds, as well as the study of Kubica-Biernat et al., which examined WNV RNA in mosquitoes from four Polish regions (including Podlaskie voivodeship), showed no presence of this pathogen. The results are consistent with other studies performed in Poland [7,9,10]. Therefore, it is highly unlikely that other tick-borne and mosquito-borne flaviviruses would induce antibodies detectable by ELISA in this study.

The TBEV disease that is limited to a peripheral flu-like phase without CNS involvement was described as abortive TBE and hypothesized to be a cause of undiagnosed seroconversions. However, in a group of 56 patients diagnosed and prospectively observed since the peripheral phase, 55 (98.2%) progressed to the neurologic phase and only one recovered after the initial febrile episode, which means that abortive TBE is rare compared to a typical biphasic disease with CNS involvement and cannot account for a large number of undiagnosed cases [17]. Thus, the seropositivity in persons with no history of TBE seems to be caused mostly by asymptomatic or very mild afebrile infections and only sporadically by an abortive form of TBE virus infection [17]. These cases cannot be diagnosed on clinical grounds and their frequency remains unknown [11]. However, they could significantly exceed the number of symptomatic cases. In a prospective study of a high risk population in Sweden, Gustafson et al. observed two cases of TBE versus eight cases of asymptomatic seroconversion, suggesting that as much as 80% of TBEV infections may not manifest clinically [15].

The reasons for an apparently high rate of asymptomatic infections with TBEV remain unclear. The virus-related factors, for example the low infectious dose or low pathogenicity of the infecting strain could contribute to the favorable outcome of the infection. However, all the subjects came from a relatively small geographic area, in which only European TBEV subtype with a high level of nucleotide sequence similarity between different isolates circulates [6], which makes any significant impact of the TBE variability unlikely. The animal models suggest that the course of the TBEV infection is determined in several stages, from the local replication and spread from the infection site, through peripheral infection and viremia, penetration across the blood–brain barrier (neuroinvasion) to the CNS involvement. Moreover, host-related factors play an important role in determining the outcome at each of these stages [49]. The ineffective or misdirected immune response may be unable to control the infection or even facilitate it, for example by virus spread within infected leukocytes or blood–brain barrier disruption by inflammatory mediators, determining a highly variable presentation, from asymptomatic to clinically severe. This in turn might depend on genetic factors, some of which have been suggested in the population-based genetic association studies comparing TBE patients to healthy individuals from the endemic area [13,50,51,52], but most probably remains undefined. We suggest that that the healthy seropositive individuals from the endemic areas, similar to those identified in our study, could constitute a highly relevant control group in future genetic association studies. Such subjects are likely to have been infected by TBEV in the past without having succumbed to a symptomatic neuroinfection, so their direct comparison with patients with symptomatic TBE would be optimal to reveal the potential genetic predisposing and protective factors.

This study has several limitations. The reported vaccination rate might be overestimated due to the nature of an anonymous survey. Second, the immunological response after the TBE infection fades over time, which might underestimate the calculated seroprevalence. Additionally, due to inability to verify the survey data and lack of access to personal health information or other identifiable data, the true rate of asymptomatic and mild TBEV infections among the seropositive individuals should be interpreted with caution.

## 5. Conclusions

Our findings confirm that exposure to ticks in northeastern Poland is associated with a high risk of TBEV infection. In addition, the rate of mild and asymptomatic infections seems to be high and the identification of infected people would be a valuable source of information that would enable a more detailed understanding of the etiopathogenesis of the disease, as well as predisposing and protective factors, both genetic and environmental. Considering the estimated high incidence of the disease in the unvaccinated population and low vaccination rates, there is an urgent need to raise awareness and knowledge about the benefits and importance of TBE vaccination. Implementing a vaccination register would be valuable for following the epidemiological situation of TBE in relation to vaccination coverage.

## Figures and Tables

**Table 1:** Characteristics of surveyed parents of children hospitalized in the Department of Pediatric Infectious Diseases in Bialystok.

Individual—Related Characteristic	Number (%)
**Gender**	**419 (100%)**
Male	74/419 (17.7%)
Female	345/419 (82.3%)
**Age**	**426 (100%)**
<29	76/426 (17.8%)
30–39	252/426 (59.2%)
40–49	85/426 (20%)
50–59	11/426 (2.6%)
>60	2/426 (0.4%)
**Education**	**426 (100%)**
Primary	11/426 (2.6%)
Secondary	134/426 (31.4%)
Higher	281/426 (66%)
**Place of residence**	**426 (100%)**
Village	175/426 (41.1%)
City more than 30,000 inhabitants	251/426 (58.9%)
**Close relative diagnosed with TBD**	104/433 (24%)
**Tick-bite anytime in the past**	270/438 (61.6%)
**Tick-bite in the last year**	75/444 (16.9%)
**Tick-bite in the respondent’s child**	139/433 (32.1%)

Data presented as frequencies and percentages. Denominators lower than the study group indicate missing data. Abbreviations: TBD, tick-borne disease.

## Data Availability

The datasets used and/or analyzed during the current study are available from the corresponding author on reasonable request.
